# A Christian Faith-Based Facebook Intervention for Smoking Cessation in Rural Communities (FAITH-CORE): Protocol for a Community Participatory Development Study

**DOI:** 10.2196/52398

**Published:** 2023-12-13

**Authors:** Pravesh Sharma, Brianna Tranby, Celia Kamath, Tabetha Brockman, Anne Roche, Christopher Hammond, LaPrincess C Brewer, Pamela Sinicrope, Ned Lenhart, Brian Quade, Nate Abuan, Martin Halom, Jamie Staples, Christi Patten

**Affiliations:** 1 Psychiatry and Psychology Mayo Clinic Health System Mayo Clinic Eau Claire, WI United States; 2 Behavioral Psychology Mayo Clinic Rochester, MN United States; 3 Center for the Science of Health Care Delivery Mayo Clinic Rochester, MN United States; 4 Health Equity and Community Engagement in Research Mayo Clinic Rochester, MN United States; 5 Psychology Mayo Clinic Rochester, MN United States; 6 Psychiatry Johns Hopkins School of Medicine Baltimore, MD United States; 7 Cardiovascular Medicine Mayo Clinic Rochester, MN United States; 8 Psychiatry and Psychology Mayo Clinic Rochester, MN United States; 9 Living Water Church Cameron, WI United States; 10 Bethesda Lutheran Church Eau Claire, WI United States; 11 Valleybrook Church Eau Claire, WI United States; 12 St John's Lutheran Church Bloomer, WI United States; 13 Renew Church Eau Claire, WI United States

**Keywords:** community, participatory, community-based participatory research, faith, smoking cessation, Facebook, social media, mobile phone

## Abstract

**Background:**

Tobacco smoking remains the leading cause of preventable morbidity and mortality in the United States, with significant rural-urban disparities. Adults who live in rural areas of the United States have among the highest tobacco smoking rates in the nation and experience a higher prevalence of smoking-related deaths and deaths due to chronic diseases for which smoking is a causal risk factor. Barriers to accessing tobacco use cessation treatments are a major contributing factor to these disparities. Adults living in rural areas experience difficulty accessing tobacco cessation services due to geographical challenges, lack of insurance coverage, and lack of health care providers who treat tobacco use disorders. The use of digital technology could be a practical answer to these barriers.

**Objective:**

This report describes a protocol for a study whose main objectives are to develop and beta test an innovative intervention that uses a private, moderated Facebook group platform to deliver peer support and faith-based cessation messaging to enhance the reach and uptake of existing evidence-based smoking cessation treatment (EBCT) resources (eg, state quitline coaching programs) for rural adults who smoke.

**Methods:**

We will use the Integrated Theory of Health Behavior Change, surface or deep structure frameworks to guide intervention development, and the community-based participatory research (CBPR) approach to identify and engage with community stakeholders. The initial content library of moderator postings (videos and text or image postings) will be developed using existing EBCT material from the Centers for Disease Control and Prevention Tips from Former Smokers Campaign. The content library will feature topics related to quitting smoking, such as coping with cravings and withdrawal and using EBCTs with faith-based message integration (eg, Bible quotes). A community advisory board and a community engagement studio will provide feedback to refine the content library. We will also conduct a beta test of the intervention with 15 rural adults who smoke to assess the recruitment feasibility and preliminary intervention uptake such as engagement, ease of use, usefulness, and satisfaction to further refine the intervention based on participant feedback.

**Results:**

The result of this study will create an intervention prototype that will be used for a future randomized controlled trial.

**Conclusions:**

Our CBPR project will create a prototype of a Facebook-delivered faith-based messaging and peer support intervention that may assist rural adults who smoke to use EBCT. This study is crucial in establishing a self-sufficient smoking cessation program for the rural community. The project is unique in using a moderated social media platform providing peer support and culturally relevant faith-based content to encourage adult people who smoke to seek treatment and quit smoking.

**International Registered Report Identifier (IRRID):**

PRR1-10.2196/52398

## Introduction

### Background and Significance

Tobacco smoking remains the leading cause of preventable morbidity and mortality in the United States, contributing to approximately 480,000 premature deaths annually [1]. This comes at a high economic cost to our society. Data from a recent national report showed that smoking-related illnesses cost the United States over US $600 billion in 2018 [2]. Evidence-based smoking cessation treatments (EBCTs) are greatly underused by people who smoke. For example, the use of state quitline coaching programs and pharmacotherapy enhances smoking quit attempts and cessation [3], but many people who smoke are unaware of or do not use state quitlines [4-6]. Schauer et al [7] analyzed data from the 2009 to 2010 National Adult Tobacco Survey and found that quitline awareness was 53.9% among people who smoke. Further, among individuals who attempted to quit smoking in the past year and had knowledge of the quitline's existence, their use rate was only 7.8%. Therefore, efforts to improve access, information, and acceptance of EBCTs are required.

Significant rural-urban disparities exist in the United States concerning tobacco smoking, smoking-related health outcomes, and smoking cessation treatment access. Adults who live in rural areas of the United States have among the highest tobacco smoking rates in the nation and experience a higher prevalence of smoking-related deaths and deaths due to chronic diseases for which smoking is a causal risk factor [8]. Trends from the last several years show that rural areas experience higher rates of smoking-related chronic diseases than their urban counterparts [9,10]. Compared to urban residents, people living in rural areas are generally older, have lower adherence to preventative screening [10-12], and face significant treatment barriers related to social determinants of health, which puts them at a higher risk of developing chronic diseases [13,14]. A lack of health care specialists in rural areas further exacerbates these barriers to seeking care [15,16]. For example, a recent study by the Centers for Disease Control and Prevention (CDC) found that people living in rural areas had lower rates of cancer diagnoses but experienced higher death rates for all cancer types when compared to people living in nonmetropolitan and metropolitan areas, highlighting rural patients’ challenges in accessing necessary health care [17]. Rural communities face tobacco-related disparities that expose them to difficulty seeking access to smoking cessation treatment support, ultimately leading to a higher prevalence of chronic diseases. For example, rural communities have a higher rate of cigarette smoking than urban areas, a trend observed for both women (rural vs urban: 25% vs 13%) and men (rural vs urban: 29% vs 19%), but tobacco control policies and cessation efforts tend to favor urban areas more than rural areas [8]. Due to difficulties such as geography, infrastructure, and corporate barriers, those living in rural areas have difficulty accessing tobacco cessation services [8].

One approach for improving access and overcoming barriers to engagement in underserved populations is through the use of digital technology and social media platforms. There has been a growing recognition that leveraging digital technology could encourage the positive behavior change necessary to quit smoking and manage chronic conditions like cancer and heart disease. By doing so, barriers that impede access to EBCT can be lessened [18]. For example, the use of social media, such as Facebook, could be used to deliver smoking cessation behavioral interventions such as motivational interviewing (MI) and peer support and encourage the use of EBCT interventions. Facebook is the most used social media platform in the United States and has a broad reach. It promotes engagement and connection among peers that can be used in smoking cessation efforts at a relatively low cost. In addition, Facebook is a helpful tool for supporting smoking cessation through online communication and sharing of experiences among users. The experience allows users and study staff to share intervention-related pictures, videos, and images to encourage discussion. This represents a distinct advantage over face-to-face approaches or web-based services [19,20]. In recent years, many studies have used social media, including Facebook, for smoking cessation interventions. However, the use of Facebook to deliver EBCT with a faith-based component developed by the community-based participatory research (CBPR) approach is lacking.

Another approach to improve access and uptake of evidence-based interventions is by culturally adapting them to fit the specific needs and contexts of individuals who underuse them. Studies have shown that the implementation of EBCT with community co-design and engagement enhances its receptivity and uptake [21]. One such effective approach is CBPR, a model that equitably involves community members, organizations, and academic researchers in all aspects of the research process. As a result, all partners can contribute their expertise with shared ownership and responsibility; knowledge gained is enhanced, and action is integrated to improve community members’ health and well-being [22]. Evidence also shows that culturally relevant messaging delivered through CBPR approaches increases [23,24] study recruitment, EBCT resources acceptance, and cessation rates [25-27]. This approach is particularly important for hard-to-reach marginalized rural populations, by empowering them to be agents of maintained change in their communities. Therefore, our study will use the CBPR strategy to center rural end users to co-design the intervention to promote its maintainability and lasting impact in the rural community.

Numerous research studies have evaluated the effect of spirituality and religious beliefs on positive health behaviors. For example, these beliefs may help to reduce stress through engagement with coping skills and lifestyle factors such as improved diet, attendance to preventive care, exercise, and reduction in alcohol drinking, and so forth, which ultimately improves cardiovascular health and general well-being [28-31]. In addition, many studies have shown a negative correlation between faith-based involvement and cigarette smoking [32-37]. Research suggests that individuals participating in faith-based activities are less likely to smoke or begin smoking in the future [37,38]. Whooley et al [37] found that nonsmoking individuals who attended religious services less than once a month were at twice the risk of starting smoking compared to those who attended services regularly over 3 years. In addition, a study conducted by Kim et al [39] among Korean men found that church attendance may assist individuals in quitting smoking due to social prohibitions from other members of the congregation. The mechanism behind this phenomenon is theorized that religious attendance offers spiritual support, an essential source of resiliency, and reduces stress [31] that would otherwise perpetuate cigarette smoking [40]. In addition, faith-based congregants may provide peer support in the smoking cessation journey [31,41]. Since religious participation offers an avenue for smoking cessation and the faith-based CBPR approach has not been thoroughly evaluated among the rural population for smoking cessation, our study attempts to fill this research gap.

Participants’ access to clinical trials in rural areas can be a challenge due to transportation barriers in traveling to clinical trial centers and the difficulties associated with spending several hours away from daily wages to participate in research studies. Therefore, participation rates in rural substance use treatment clinical trials are low. Decentralization of clinical trials using digital tools, as we propose in this study, could mitigate this problem.

### Study Aims and Objectives

In this report, we describe a study protocol focused on developing and beta testing an innovative faith-based Facebook intervention for rural smokers. The overall objective of this study is to develop a private, moderated, faith-based Facebook group intervention platform using peer and pastoral support for rural people who smoke to enhance the use and reach of existing EBCT resources.

Our study-specific aims are (1) to describe the process we will use to develop a faith-based Facebook group intervention for smoking cessation that promotes access to and use of EBCT using the CBPR approach and (2) to assess the preliminary feasibility and uptake of the intervention with a 1-month beta test.

## Methods

### Theoretical Framework

**Figure 1:**
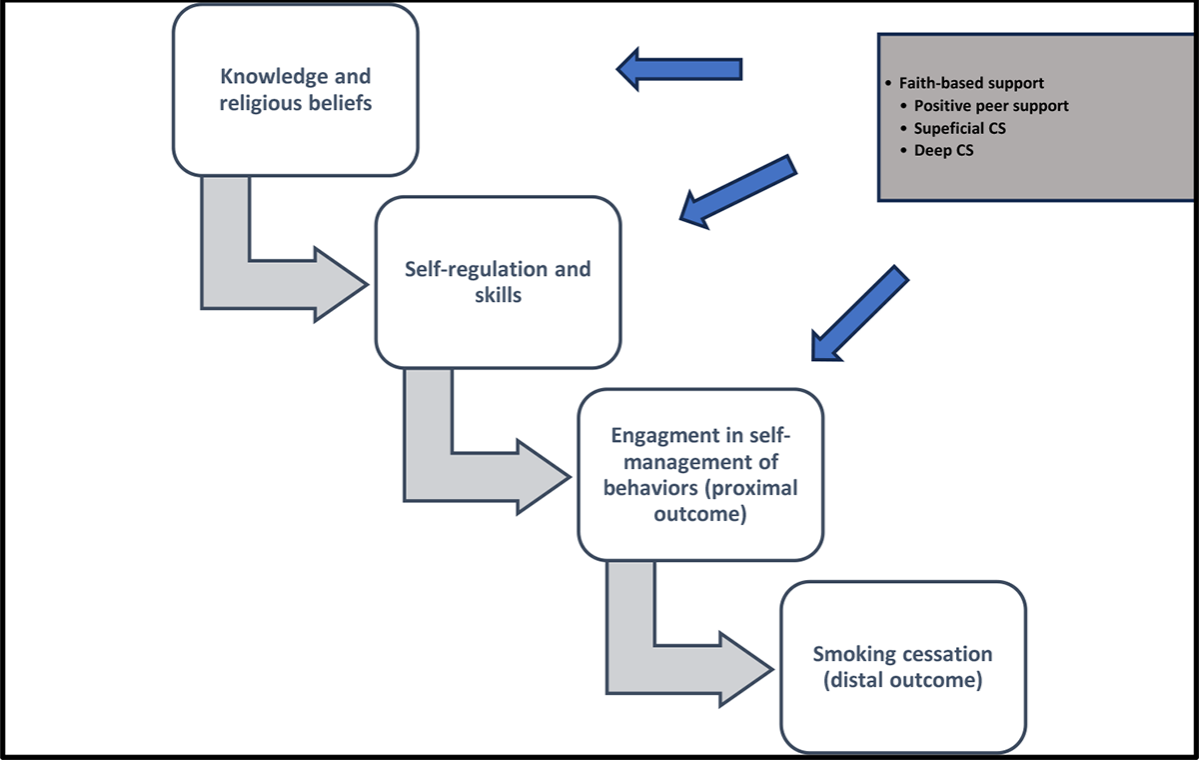
Theoretical framework. CS: cultural sensitivity.

We will use the Integrated Theory of Health Behavior Change (ITHBC) [42] and surface or deep structure [43] frameworks as guidance for intervention development (Figure 1). According to the ITHBC framework, an individual is more likely to engage in a behavior change when they understand the disease-related risk factors they are indulging in and if treatment recommendations (including minimizing risk factors) align with their personal beliefs. Therefore, the content of our intervention will be evidence-based (EBCT in this case) and will be prepared to increase the participants’ knowledge about chronic diseases and their risk factors, such as smoking, to enhance their self-efficacy, outcome expectations, and goal congruence.

Self-regulation, a second key factor in making a behavior change, may be enhanced through increased knowledge and reflection on one’s beliefs. Self-regulation involves setting goals, monitoring, reflecting, decision-making, planning, execution, self-evaluation, and managing emotions during change. We will design intervention content to facilitate self-regulation through Facebook posts aimed at increasing knowledge about smoking cessation content (eg, thoughts, intentions, and actions) in congruence with faith-based beliefs.

The third construct, social facilitation, involves social support that encourages behavior change. Participants will receive supervised social support through Facebook engagement with peers on the same journey of smoking cessation. Before the desired outcome (distal outcomes) of smoking cessation, the individual will engage in several self-management behaviors (proximal outcomes), such as calling the state quitline and engaging in Facebook conversations and posts that take them to their final or distal outcome of smoking cessation. For this study, we plan to develop an intervention that will target proximal outcomes (distal outcomes not the scope of this study) of self-management behaviors, measured by actions such as calling a quitline and engaging in Facebook conversations and posts. As noted earlier, since proximal outcomes are influenced by an individual’s knowledge, beliefs, and self-regulation skills, we will use evidence-based material and faith-based messages to facilitate proximal outcomes.

To ensure our messages are culturally aligned and faith-based, we will use the surface and deep structure framework [44,45]. Surface structure considers observable social and behavioral characteristics of participants, such as race, gender, and family structure, while deep structure takes into account cultural beliefs and values. Finally, we will use a planning framework based on the CDC recommendations for developing social media and other digital health communication tools [46] to guide the critical content components of Facebook messages, consistent with behavioral therapy.

### Study Design

The study will use the CBPR approach and will be conducted at Mayo Clinic Health System, Eau Claire, Wisconsin. Before the start of the study, several small-group in-person and online meetings were held in the community with faith-based leaders of local churches to gauge their interest in forming a partnership and brainstorming the study idea. The local faith-based leaders agreed to collaborate and participate in the review of the study design and intervention materials and provide general input on faith-based topics. The pastors were informed that they will be part of the “core research team” and will approve documents including manuscripts, flyers, and other materials before publication and community dissemination. Our other partners for this study included the Mayo Clinic Center for Clinical and Translational Science (CCaTS), community engagement and rural health programs, and the Center for Health Equity and Community Engagement Research (CHCR) that will facilitate the formation of a community advisory board (CAB) and engage the services of the community engagement (CE) studio to get community feedback on the development of the intervention.

### Recruitment

#### Community Advisory Board

The CAB will consist of 10-12 individuals from the community. We will ensure that the board comprises diverse individuals from different sexes, races, and age groups with fair representation. Print and e-flyers highlighting roles and responsibilities will be used to facilitate recruitment. The flyers will be posted on the Mayo Clinic display board and emailed or mailed to the community partner organizations, including church partners. CAB members will receive an honorarium of US $150 per meeting.

#### CE Studio

CHCR staff will assist in recruiting CE studio members, consisting of community members who represent end users of the intervention. Advertisement flyers will be used to facilitate the recruitment. The CE studio will consist of 6-8 rural community experts who will join a 1-time 90-minute online meeting to give feedback on the content of smoking cessation material for the faith-based Facebook intervention. Eligibility criteria for participation are as follows: (1) aged >18 years, (2) lives in the rural area of Wisconsin or Minnesota, (3) smoked at least 1 cigarette per day over the past 7-day period, and (4) Christian faith. Community experts will receive a US $50 visa card for their time.

#### Beta Test

A Facebook group size of 10 was the minimum number for optimal engagement in previous studies [47,48]. Thus, we will beta test the Facebook group with an estimated purposeful sample of 15 individuals who currently smoke and attempt to ensure that the sample comprises diverse individuals from different sexes, races, and age groups with fair representation. A 1-page information sheet or flyer codeveloped with the core research team and CAB will be posted on the Mayo Clinic display board and emailed or mailed to the community partner organizations, including local church partners. Study staff will administer an initial set of screening questions over the phone to screen for eligibility.

The eligibility criteria for participation are (1) aged >18 years; (2) smoked at least 1 cigarette per day over the past 7-day period; (3) have access to the internet on a mobile phone, computer, or tablet; (4) willing to create a Facebook account and willing to participate in the Facebook intervention for 1 month; and (5) either self-identify as a person of Christian faith or recognize that study posts will include Christian faith-based content, including Bible verses. Ineligible individuals (positive for smoking but unwilling to engage with Facebook intervention) will receive EBCT referral information via postal mail (printed materials) or email. The material will include information on their state tobacco quitline. Participants will also receive information on the National Cancer Institute’s free population-level smokefree.gov website resources. The website, tailored to all readiness levels to quit smoking, includes a quitting guide, texting program, smartphone apps, online live chat, URL, and a toll-free number (1-800-QUIT-NOW) for state quitlines [26].

### Procedures

#### Phase 1: Intervention Development

Our research team is comprised of rural health, substance use, and health disparities researchers, pastors from local churches, and rural patient advocates from the Mayo Clinic enterprise. We will compile the initial items for the content library by drawing on empirical research, existing literature, and our research team’s combined clinical research and pastoral experience. We will use existing EBCT videos and other content postings from the CDC Tips from Former Smokers Campaign that promote the use of EBCTs to various disparate groups, including rural people who smoke. The preliminary content library will feature various topics related to quitting smoking, such as dealing with stress, nicotine withdrawal, cravings, personal health, and culturally relevant activities that can help smokers quit associated with relevant faith-based messages or bible quotes, for example, how prayer, faith, connection to others, grace, temptation, patience, forgiveness, and so forth, can support with the use of evidence-based smoking cessation intervention. The content topic will also discuss motivations for quitting, like the importance of family and preventing relapse, and encouraging EBCT, such as using state quitlines and pharmacotherapy. Each topic will have corresponding Facebook posts in text and embedded videos and images. Further, for each topic of content, a sample of the supplementary text is provided for Facebook group moderators (who will oversee the posting and engage participants in conversation) and a probe for generating further discussion or ways to respond to users’ questions. Facebook group moderators will select posts based on ongoing group conversations and respond to individual posts and queries while posting for the entire group.

#### Phase 2: Community Members’ Feedback

**Figure 2:**
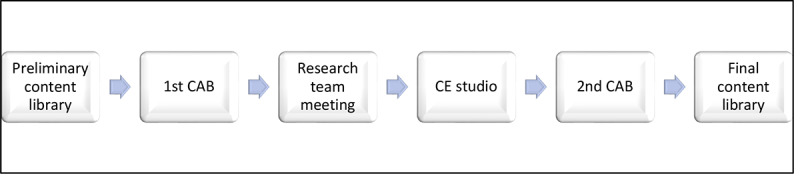
Community engagement plan for the development of the content library. CAB: community engagement board; CE: community engagement.

The preliminary content library will be evaluated for content validity through the guidance and advice obtained from CAB members and a CE studio [49,50]. The community feedback process consists of 3 critical steps. First, we will conduct a CAB meeting to gather feedback and input on the preliminary content library. The core research team will use this feedback to revise the content library further. Second, the CE studio will be used to obtain additional feedback on the revised content library. Finally, we will conduct the second and final CAB meeting, where we will thoroughly discuss the feedback provided by the CE studio and how we implemented feedback from the first CAB meeting. Their consolidated feedback will be incorporated with input from the core research team, and the content library will be considered final (Figure 2).

#### Community Advisory Boards

Each CAB member will receive the meeting agenda, an electronic version of the preliminary content library, and the Zoom (Zoom Video Communications) meeting link in preparation for the online meeting. The online CAB meeting will last for approximately 90 minutes and will be recorded. During the meeting, the study’s principal investigator, PS, will summarize the study and facilitate introductions among the attendees. In addition, PS will present each topic within the preliminary content library and facilitate the discussion. During the discussion, each topic will be displayed, and members will provide feedback on the selected images or video, the bible quotes and their alignment with the associated Facebook post, and overall post understandability and relevance to the topics. Study staff (BT and CK) will take detailed meeting notes. The CAB members will also have an opportunity to provide open-ended feedback.

#### CE Studio

The CE studio will be a 90-minute online meeting facilitated by the CHCR staff. A CHCR staff will begin the meeting with introductions, and then PS will provide a 10 to 15-minute presentation of the study, and a CHCR staff will facilitate the discussion. Similar to the CAB, each topic will be displayed during the discussion, and community experts will provide feedback on the selected images or video, the bible quotes and their alignment with the associated Facebook post, and overall post understandability and relevance to the topics. A CHCR staff will take detailed notes and provide a summary at the end of the CE studio.

#### Phase 3: Prototype Development and Moderator Training

The Mayo Clinic Social Media Department will assist in creating a study-related Facebook page. The group will be private, hidden, and moderated to facilitate confidentiality. By this stage, the content library will be finalized, and additional postings, including Bible verses and images, will be added on an as-needed basis based on insights and feedback from CBPR processes. The moderators’ interactions with group members are critical for sparking conversation among the group members. As a result, we will choose moderators who have knowledge of the Christian faith and are familiar with Bible verses. Given that our research team includes pastors from local churches, they will be consulted on an ongoing basis by the moderators if any guidance is required. The moderators will receive comprehensive training to ensure they possess the knowledge and skills for effective group moderation. The training will cover the principles of moderation, including the best ways to engage participants, promote appropriate conversations, and redirect engagement [51,52]. They will also be trained to handle challenging situations that may arise in a web-based group. Additionally, the moderators will participate in didactic training to learn basic communication principles, such as active listening, MI, and cognitive behavioral therapy. The ultimate goal is group participant interaction and partaking. Therefore, moderators will be encouraged to help generate daily interaction among members and not just post the content daily. Moderators will post 1 selection from the intervention content library every 1-2 days. In order to stimulate engagement from participants, the moderators will reply to the posts with 1-2 prompt questions (included in the content library) directed at specific individuals and using MI techniques. They will check the group several times daily to engage with participants and address inappropriate content or misinformation.

#### Phase 4: Beta Test

**Figure 3:**
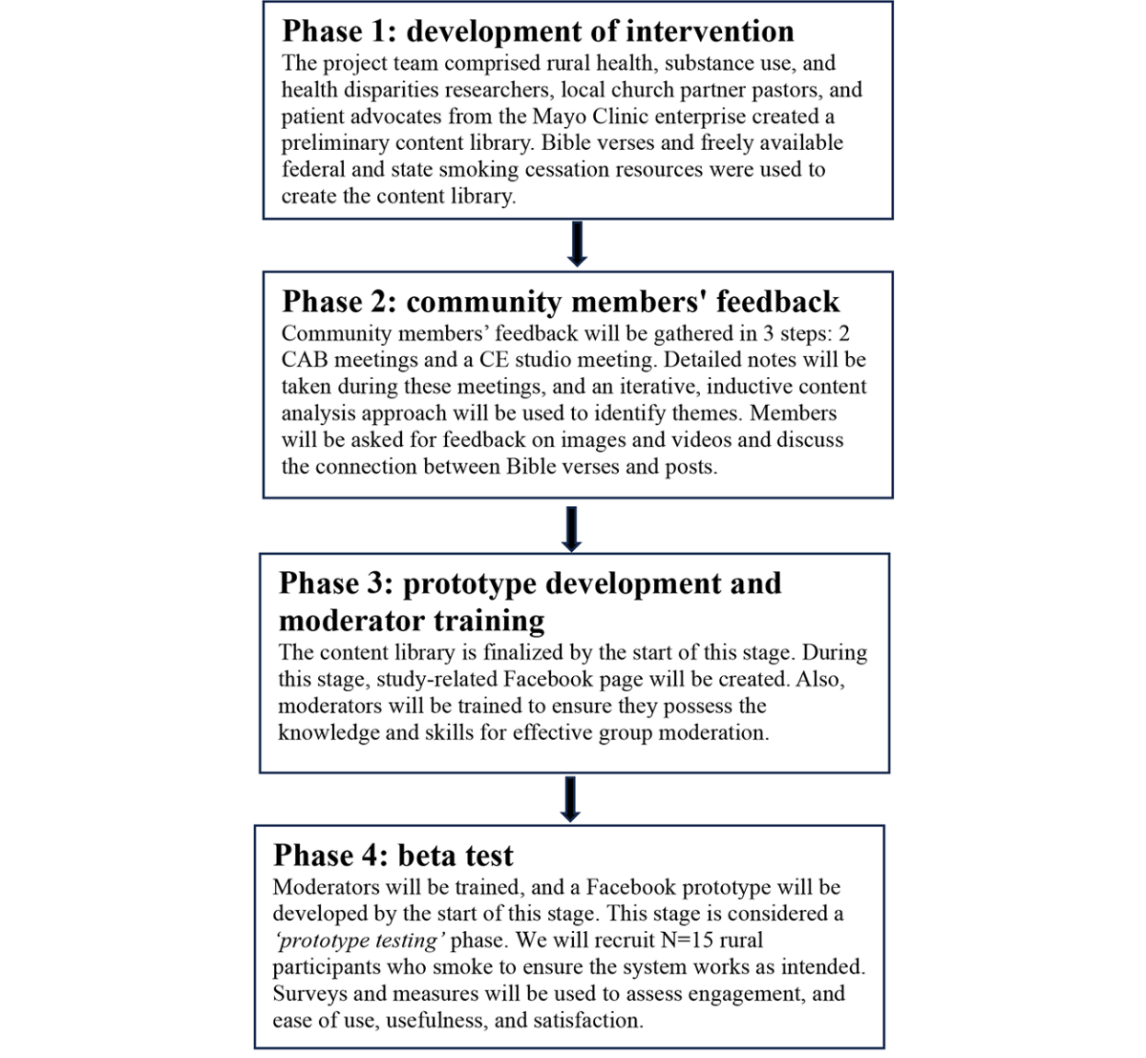
Overview of the process to develop faith-based Facebook intervention prototype and beta test to help rural people quit smoking. CAB: community advisory board; CE: community engagement.

After completing baseline assessments, eligible participants will be emailed an invite link to the intervention Facebook group. Participants will be given instructions on how to register on Facebook. Those who already have a Facebook account can either create a new user profile or use the existing one to join the group. The research staff will ensure that the participants enroll in the group within 24 hours of receiving the link. If participants experience technical difficulties, the staff will assist them. The smokefree.gov resources will also be mailed to the participants. This phase will expose participants to 30 days of moderator postings and obtain feedback to ensure the system works as intended. In addition, we will conduct a preliminary assessment of feasibility and intervention uptake (engagement) to facilitate any needed refinements prior to future randomized controlled trial (RCT) (Figure 3).

### Measures

#### Overview

For aim 2, the beta testing phase, we will use an internet-based survey to measure the following. The participants in this study will receive a survey via email through REDCap (Vanderbilt University). The survey will be open for 7 days, and participants will receive 2 reminders to complete it. The email address on file is required for this process.

#### Baseline Smoking Characteristics

We will assess the average number of cigarettes per day, the age when the person started smoking, pack-years of smoking, the number of days the person smoked in the past 30 days [53], readiness to quit using the contemplation ladder [54], the average number of quit attempts in the past 30 days, and other commercial tobacco and nicotine product use.

#### Baseline Sociodemographic Characteristics

Sociodemographic measures selected from the PhenX Toolkit assessed age, sex, gender, ethnicity, race, education, employment status, and marital status [55].

#### Study Recruitment and Retention

Recruitment feasibility measures include the number of potential participants screened, the number of eligible participants, and the number of enrolled participants from the eligibility pool. Additionally, study retention will be evaluated by determining the proportion of participants who complete postintervention assessments.

#### Treatment Satisfaction

The satisfaction with the Facebook intervention will be measured in three domains: (1) perceived ease of use, (2) usefulness, and (3) satisfaction by using the Usefulness, Satisfaction, and Ease of Use (USE) questionnaire. The scale contains 13 items and participants will rate these items on a 5-point Likert scale, ranging from 1 (strongly disagree) to 5 (strongly agree). We will examine the Facebook intervention engagement patterns using Facebook metrics and qualitatively explore common topics that emerged in participant postings [56]. Additionally, we will include open-ended questions to gather more detailed feedback from users.

#### Intervention Uptake or Engagement

In order to measure the adoption of our intervention, we will use Facebook’s objective engagement metrics such as views, reactions (such as likes and loves), comments, posts, and poll votes (where participants can make multiple selections). By calculating the total number of times participants engaged with the intervention, we can accurately assess its uptake.

### Statistical Analysis

Aim 1 will have 2 CAB meetings and 1 CE studio session. Members will be asked to provide helpful feedback on the content's flow, understandability, and relevance. Detailed notes will be taken during these meetings, and an iterative, inductive content analysis approach will be used to identify themes. A total of 2 investigators will work together to code the responses, and a third investigator will resolve any disagreements until a consensus is reached. Aim 2 will summarize sample characteristics, social media usability measures, and most and least preferred concepts using descriptive statistics, including means, percentages, and frequencies. Open-ended responses will be summarized, and themes will be generated using content analysis as described in aim 1.

### Missing Values

Because assigning missing data as smoking is not necessarily a conservative approach [57], we will also explore multiple imputation methods [58,59] to classify lost to follow-up as cigarette smokers or nonsmokers and conduct sensitivity analyses as appropriate.

### Ethical Consideration

The study was deemed exempt by the Mayo Clinic Institutional Review Board (23-000837). The CAB members will receive a US $150 honorarium. CE studio community experts will receive US $50. The participants will receive US $25 for completing the baseline assessment and US $25 for the postintervention assessment. For the beta test phase, study data will be deidentified.

## Results

The results of this project will lead to the development of a faith-based smoking cessation content library. In addition, we are planning a pilot 2-arm parallel group, RCT, which will include evaluation of control (mailed materials and smokefree.gov information) versus Facebook intervention (information mailed to the control group plus Facebook intervention developed during this study) among diverse populations.

## Discussion

### Principal Findings

Our study focuses on collaborating with community stakeholders to co-design a faith-based, peer-supported Facebook-delivered intervention prototype to address smoking cessation among rural people who smoke. This study is essential to a self-maintaining smoking cessation program for the rural population. We believe that Facebook group moderation is an important component of this program to increase participant engagement and provide behavioral support and access to cessation treatment resources and service linkages when required. In addition, the supervised Facebook group may lead to better peer support among participants who can learn from each other’s experiences and share their smoking cessation journey.

No such Facebook group exists that uses faith-based support as a component and encouragement to use EBCTs such as quitlines and cessation pharmacotherapy. During the preliminary meetings, our community stakeholders have shown interest in being trained as moderators and ensuring the continuity of the proposed Facebook group so that the intervention is self-maintained within the community once the study is over.

### Limitations

Our sample is restricted to the Midwest population and stakeholders, which may limit the generalizability to other rural populations. However, this phase is focused on prototype development, and future pilot study steps will pave the way toward larger, more generalizable evaluations of digitally enhanced interventions. Our study will recruit individuals with access to the internet and smart devices to access Facebook, which may lead to selection bias. For future studies, investigators may consider providing a loaner digital device to participants with no digital access to eliminate this selection bias.

### Conclusions

Our study will provide preliminary steps, including possible challenges before the RCT for a faith-based, Facebook-delivered EBCT intervention for rural populations that smoke. Our study has a broader interest in faith-based stakeholders and potentially be self-maintainable within the community. This approach can potentially support rural communities by granting them access to community-based health care that is maintainable. Our study design has the capability to provide a tailored online intervention with fidelity. Furthermore, our study uses existing federal and state EBCTs, using available resources to aid in smoking cessation.

## Abbreviations

CAB: community advisory board
CBPR: community-based participatory research
CCaTS: Center for Clinical and Translational Science
CDC: Centers for Disease Control and Prevention
CE: community engagement
CHCR: Center for Health Equity and Community Engagement Research
EBCT: evidence-based smoking cessation treatment
ITHBC: Integrated Theory of Health Behavior Change
MI: motivational interviewing
RCT: randomized controlled trial
USE: Usefulness, Satisfaction, and Ease of Use
